# Diabète gestationnel révélé par une acidocétose diabétique inaugural: à propos de un cas

**DOI:** 10.11604/pamj.2017.27.148.12155

**Published:** 2017-06-29

**Authors:** Serge Irie Bi Gohi, Pete Yaich, Koffi N'guessan, Bernard Ogondon, Yapo Brouh

**Affiliations:** 1 Service d'Anesthésie-Réanimation du Centre Hospitalier Universitaire de Bouaké, Côte d'Ivoire

**Keywords:** Acidocétose, grossesse gestationnel, pronostic maternel, fœtal, Ketoacidosis, pregnancy, gestational diabetes, maternal, foetal prognosis

## Abstract

L'acidocétose compliquant un diabète gestationnel est une affection rare, responsable d'une mortalité materno-fœtale importante. C'est une urgence métabolique aigue dont la prise en charge est multidisciplinaire. La précocité du diagnostic et du traitement conditionnent le pronostic vital de la mère et du fœtus. Nous rapportons l'observation d'une gestante de 27 ans à terme, avec un antécédent familial de diabète, qui a été admise aux urgences obstétricales pour un trouble de la vigilance associé à une dyspnée. Le diagnostic d'acidocétose inaugural décompensée par un paludisme grave sur un diabète gestationnel a été retenu sur la base de l'anamnèse, de l'examen clinique et du bilan paraclinique. Elle a bénéficié d'une insulinothérapie, d'une réhydratation, d'une correction des troubles ioniques et d'une traitement antipaludique. Une césarienne a été réalisée en urgence sous anesthésie générale et a permis d'extraire un macrosome de sexe masculin mort-né macéré. L'évolution a été favorable avec une reprise de la conscience et une normalisation des paramètres biologiques.

## Abstract (en)

Ketoacidosis complicating gestational diabetes is rare and responsible for severe maternal-fetal mortality. It is an acute metabolic emergency whose management is multidisciplinary. Early diagnosis and treatment affect the vital prognosis of both the mother and the fetus. We report the case of a 27-year old pregnant woman at term, with a family history of diabetes, admitted to the emergency obstetric care with alertness problems associated with dyspnoea. The diagnosis of inaugural ketoacidosis decompensated due to severe malaria associated with gestational diabetes was retained on the basis of patient's medical history, of clinical examination and paraclinical assessment. The patient received insulin therapy, rehydration therapy, correction of electrolyte imbalance as well as antimalarial treatment. She underwent emergency cesarean section under general anesthesia and a dead-born macrosome macerated male fetus was extracted. Patient's evolution was favorable, with return of consciousness and standardization of biological parameters.

## Introduction

Selon l'organisation Mondiale de la Santé (OMS), «le diabète gestationnel est un trouble de la tolérance glucidique conduisant à une hyperglycémie de sévérité variable, débutant ou diagnostiqué pour la première fois pendant la grossesse, quels que soient le traitement nécessaire et l'évolution dans le post-partum» [1]. C'est un trouble métabolique qui apparaît en fin de grossesse, qui est en relation avec une augmentation de la résistance à l'insuline et/ou un déficit de sécrétion d'insuline et qui disparaît à la naissance de l'enfant [2]. Il concerne 1 à 14% de toutes les grossesses et d'une façon générale, les patientes qui ont eu un diabète gestationnel développent dans l'avenir un autre diabète qui le plus souvent est de type 2 [3, 4]. La survenue d'une acidocétose au cours d'une diabète gestationnel est un évènement rare [5]. Nous rapportons l'observation d'une gestante qui a présenté un diabète gestationnel révélé par une acidocétose diabétique inaugural.

## Patient et observation

Mme X, âgée de 32 ans, gestante de 37 semaines d'aménorrhée (SA), deuxième geste, primipare a été admise aux urgences obstétricales pour trouble de la conscience, dyspnée et fièvre. L'interrogatoire a retrouvé une fièvre associée à des courbatures et à des céphalées survenues 7 jours avant son admission. Elle a consulté un centre de santé urbain où elle a bénéficié d'une traitement antipaludique. L'évolution sous ce traitement a été marquée par la persistance de la fièvre et par l'apparition d'une polydipsie associée à une polyphagie, une polyurie, des vomissements et des douleurs abdominales diffuses. Devant ces signes aucun traitement n'a été administré. La survenue d'une dyspnée et d'une obnubilation, a motivé son admission aux urgences obstétricales du Centre Hospitalier et Universitaire (CHU) de Bouaké. On retrouvait dans le carnet de maternité 4 consultations prénatales (CPN) de bonne qualité, un antécédent familial de diabète (tante diabétique de type 2), un antécédent personnel d'avortement tardif (5e mois de grossesse) et un bilan prénatal normal. Le poids avant la grossesse était de 90kg pour une taille de 1.69m soit un indice de masse corporelle à 31.5kg/m^2^. L'examen physique à l'admission a retrouvé une patiente obnubilée avec un score de Glasgow à 10. La fréquence respiratoire était à 38 cycles/mn, la fréquence cardiaque à 125 battements/mn, la pression artérielle à 110/60 mm/Hg, la température à 39^o^ C avec des signes de déshydratation globale. L'examen obstétrical notait une absence des mouvements actifs fœtaux (MAF). L'échographie obstétricale réalisée en urgence a permis de confirmer la mort fœtale in utero avec présence d'une hydramnios de 3 litres. Au plan biologique, la glycémie veineuse était à 5.45 g/l avec une glycosurie et une cétonurie à plus de quatre croix à la bandelette réactive. La goutte épaisse était positive à 3000 trophozoites/mm^3^ et la numération formule sanguine (NFS) notait une absence d'hyperleucocytose (GB: 9,36.103 /uL), un taux d'hémoglobine à 16.9g/dl, un hématocrite à 55% et les plaquettes à 236.10^3^/uL. L'ionogramme sanguin a retrouvé une hyponatrémie à 127mEq/l, une hypokaliémie à 3mEq/l et une chlorémie à 138mEq/l. On notait une cytolyse hépatique (TGO:214UI/L et TGP:121UI/L).

L'examen cytobactériologique des urines était normal avec une absence de protéinurie. Le diagnostic d'acidocétose diabétique associé à un paludisme grave type neurologique avec une mort fœtale in utero a été posé immédiatement en milieu obstétrical. La patiente a été transférée en réanimation pour prise en charge thérapeutique. Les gaz du sang ont mis en évidence une acidose métabolique (pH = 7.15; PaO_2_ = 90mmHg; PaCO_2_= 30mmHg et bicarbonates= 15 mEq/l). L'osmolarité calculée était à 327 mmoles. Le bilan de coagulation réalisé est revenu normale. La prise en charge en soins intensifs comportait une insulinothérapie par seringue autopulsée à 10Ul/h au début avec une modulation de la vitesse selon l'évolution des glycémies, un traitement antipaludique par l'artésunate, une réhydratation et une oxygénothérapie 6l/mn au masque. La prise en charge obstétricale a consisté en la réalisation d'une césarienne faite sous anesthésie générale qui a permis d'extraire un nouveau-né masculin mort-né macéré avec un poids de naissance à 3950g. L'évolution clinique et biologique a été rapidement favorable à J2 d'hospitalisation, avec une normalisation du pH, normalisation des glycémies, disparition de la cétonurie et de la glycosurie. La patiente a été par la suite transférée à J5 d'hospitalisation en diabétologie pour la poursuite de sa prise en charge et son éducation thérapeutique. Après l'extraction du fœtus, La patiente n'a plus eu besoin d'insuline et le test de tolérance au glucose est revenu normal à 6 semaines du post-partum.

## Discussion

Depuis le dépistage systématique du diabète et la surveillance accrue des grossesses, l'incidence de l'acidocétose diabétique a nettement diminué (22% en 1976-81 et 2% en 1985-95 [6]). La prévalence de nos jours est estimée à 1 à 3% des grossesses [7]. Cependant, l'acidocétose sur grossesse est une urgence métabolique aiguë, qui met en danger aussi bien la mère que le fœtus [7, 8]. Elle survient généralement en fin de grossesse et concerne essentiellement les patientes diabétiques de type 1, inaugurant parfois le diabète. L'acidocétose est le mode de révélation d'une tiers des cas de diabète de type 1, touchant essentiellement des enfants ou sujets jeunes sans surcharges pondérales [6]. Toutefois dans de rare cas comme dans notre observation, cette complication peut s'observer au cours d'une diabète de type 2, voire d'une diabète gestationnel, notamment lorsque la femme suit un traitement par les corticostéroïdes ou par les bêta-2 mimétiques [6]. Notre patiente ne suivait pas de traitement par les corticostéroïdes ou par les bêta-2 mimétiques mais présentait certains facteurs de risques susceptibles de lui faire développer un diabète gestationnel. Ces facteurs étaient: l'âge (> 25 ans), obésité (IMC), antécédentfamilial de diabète et d'avortement tardif. Aussi la normalisation de la glycémie de notre patiente, après l'accouchement et l'arrêt du traitement antidiabétique étaient des arguments en faveur d'une diabète gestationnel. La survenue d'une acidocétose au cours du diabète gestationnel est un évènement rare [5].

Dans la littérature médicale, seulement 3 cas ont été rapportés [9–11]. Du point de vue physiopathologique, la grossesse est une situation diabétogène caractérisée par un état de résistance à l'insuline du fait de certaines hormones nécessaires à la croissance du fœtus (human placental lactogen, progestérone, cortisol et prolactine). Au cours de la grossesse normale, l'insulinorésistance physiologique est contrebalancée par un hyperinsulinisme réactionnel: l'équilibre glycémique est maintenu. Chez certaines femmes, la fonction pancréatique est déficiente. Il y a une altération du fonctionnement des cellules 3 du pancréas. Au fur et à mesure que l'insulinorésistance s'installe, le pancréas est de plus en plus sollicité. En fin de grossesse, lorsque la résistance à l'insuline est majeure, le pancréas est complètement dépassé et ne peut plus répondre à la demande croissante en insuline. L'insulinosécrétion est donc insuffisante pour répondre aux besoins de l'organisme, notamment en période postprandiale. Il en résulte alors, une diminution de la captation tissulaire du glucose, une augmentation de la production de glycogène par le foie et une élévation de la glycémie: on parle alors de diabète gestationnel [2, 12]. Après une hyperglycémie provoquée par voie orale (HGPO), chez les femmes présentant un diabète gestationnel, la réponse insulinique par unité de stimulus glycémique est beaucoup plus faible que lors d'une grossesse normale. Par ailleurs, le pic plasmatique d'insuline apparaît beaucoup plus tardivement [2, 12]. L'utilisation du glucose maternel par le fœtus est responsable de l'augmentation de production d'acides gras libres et de corps cétoniques hépatiques par la mère [13]. Les nausées et vomissements maternels favorisaient la déshydratation. Le passage des corps cétoniques à travers la barrière placentaire est responsable d'une hypoxie à l'origine d'une souffrance fœtale, pouvant évoluer vers la mort in utero [13]. Selon certains auteurs, la mortalité fœtale au cours de l'acidocétose est élevée de l'ordre de 40 à 100% et corrélée à la gravité de la décompensation acidocétosique (taux de glucose élevé, présence d'une coma) [14, 15].

Les facteurs de révélation du diabète et de déclenchement de l'acidocétose peuvent être infectieux, médicamenteux ou toxiques [16]. Chez notre patiente, le facteur déclenchant retrouvé était un paludisme grave. Cette constatation était en accord avec celle faite par Lokrou qui retrouvait la prépondérance du paludisme comme facteur déclenchant (33% des causes infectieuses) de l'acidocétose de l'adulte en Côte d'ivoire [17]. Les 3 cas rapportés dans littérature, avaient également une étiologie infectieuse (infection urinaire, bronchopneumopathie) comme facteur déclenchant [9–11]. Aussi, ces patientes avaient une conscience normale à leur admission en unités de soins intensifs. Ce qui n'était pas le cas de notre patiente qui a été admise dans un état de coma à l'hôpital. Ce coma était lié à une négligence et un retard à la consultation malgré les signes annonciateurs du diabète. Lokrou retrouvait un délai d'évolution de la symptomatologie avant la prise en charge de l'acidocétose de 19 ± 23.2 jours [17]. L'acidocétose sur diabète gestationnel est une urgence métabolique aigue dans laquelle le pronostic materno-fœtale est conditionné par un diagnostic et une prise en charge précoce. Selon les cas rapportés dans la littérature, l'évolution n'a été défavorable avec mort fœtale in-utero chez une gestante qui avait consulté 6 jours après le début des symptômes [9–11]. Ce constat était identique avec celui de notre patiente qui avait été admise 7 jours après le début des symptômes et présenté une mort fœtal in utero. Le traitement et la surveillance sont identiques à ceux mis en place chez la femme non enceinte avec instauration d'une surveillance fœtale par monitoring. La prévention constitue un élément primordial. Elle repose sur un dépistage systématique du diabète gestationnel, dès le 2^e^ et 3^e^ trimestre de grossesse de toute les patientes présentant des facteurs de risque et une surveillance accrue (obstétricale et diabétologique) des grossesses. En effet, grâce à la surveillance étroite des grossesses l'incidence de l'acidocétose diabétique a nettement diminué (22% en 1976-81 et 2% en 1985-95) [6] La mortalité apparaît très élevée dès lors que le diagnostic est tardif [7].

## Conclusion

L'acidocétose diabétique compliquant un diabète gestationnel est une pathologie rare. C'est une affection grave dont le diagnostic à un stade tardif peut s'avérer létale pour la mère et le fœtus. La prévention constitue un élément primordial. Le dépistage systématique de toute les patientes à risques entre la 24^ème^ et 28^ème^ semaine de gestation par un test de screening (test de o'sullivan) permettra de diagnostiquer le diabète gestationnel et traiter davantage de femmes.

## Conflits d'intérêts

Les auteurs ne déclarent aucun conflit d'intérêts.
